# Rapid Detection and Quantification of Novel Psychoactive Substances (NPS) Using Raman Spectroscopy and Surface-Enhanced Raman Scattering

**DOI:** 10.3389/fchem.2019.00412

**Published:** 2019-06-19

**Authors:** Howbeer Muhamadali, Alexandra Watt, Yun Xu, Malama Chisanga, Abdu Subaihi, Carys Jones, David I. Ellis, Oliver B. Sutcliffe, Royston Goodacre

**Affiliations:** ^1^Department of Biochemistry, Institute of Integrative Biology, University of Liverpool, Liverpool, United Kingdom; ^2^School of Chemistry, Manchester Institute of Biotechnology, University of Manchester, Manchester, United Kingdom; ^3^Department of Chemistry, University College in Al-Qunfudah, Umm Al-Qura University, Mecca, Saudi Arabia; ^4^MANchester DRug Analysis and Knowledge Exchange, Faculty of Science and Engineering, School of Science and the Environment, Manchester Metropolitan University, Manchester, United Kingdom

**Keywords:** spectroscopy, raman, SERS, drug detection, psychoactive compounds

## Abstract

With more than a million seizures of illegal drugs reported annually across Europe, the variety of psychoactive compounds available is vast and ever-growing. The multitude of risks associated with these compounds are well-known and can be life threatening. Hence the need for the development of new analytical techniques and approaches that allow for the rapid, sensitive, and specific quantitative detection and discrimination of such illicit materials, ultimately with portability for field testing, is of paramount importance. The aim of this study was to demonstrate the application of Raman spectroscopy and surface-enhanced Raman scattering (SERS) combined with chemometrics approaches, as rapid and portable techniques for the quantitative detection and discrimination of a wide range of novel psychoactive substances (methcathinone and aminoindane derivatives), both in powder form and in solution. The Raman spectra of the psychoactive compounds provided clear separation and classification of the compounds based on their core chemical structures; *viz*. methcathinones, aminoindanes, diphenidines, and synthetic cannabinoids. The SERS results also displayed similar clustering patterns, with improved limits of detections down to ~2 mM (0.41 g L^−1^). As mephedrone is currently very popular for recreational use we performed multiplexed quantitative detection of mephedrone (4-methylmethcathinone), and its two major metabolites (nor-mephedrone and 4-methylephedrine), as tertiary mixtures in water and healthy human urine. These findings readily illustrate the potential application of SERS for simultaneous detection of multiple NPS as mixtures without the need for lengthy prior chromatographic separation or enrichment methods.

## Introduction

Novel Psychoactive Substances (NPS) are defined as any substance able to affect a person's mental capability or emotional state (Welter-Luedeke and Maurer, 2016). They have been reported in the earliest of human records, used by healers in primitive medicines with compounds like opium, by priests in religious ceremonies, and in the general population for recreational use with drugs such as nicotine and caffeine.

According to the 2018 report from the United Nations Office on Drugs and Crimes, around 275 million people worldwide have used drugs at least once during 2016, and around 167,750 deaths directly due to drug use have been registered in 2015 (UNODC., 2018). The emergence of hundreds of NPS into various markets, with little to no understanding of their toxicity, pharmacodynamics, and long-term side effects (Tracy et al., 2017), has created a challenge for their detection, identification and quantification, as well as treatment and control efforts. Despite the availability of various detection methods such as: capillary electrophoresis (Nguyen et al., 2015; Saar-Reismaa et al., 2018), immunoassays (Poklis et al., 1993), nuclear magnetic resonance (NMR) spectroscopy (Balayssac et al., 2014), and DNA aptameric sensors (Rauf et al., 2017), the current gold standard techniques for the analysis of such materials are so-called hyphenated methods. These typically involve a combination of prior chromatographic separation techniques coupled to mass spectrometry and include gas or liquid chromatography mass spectrometry (GC-MS, LC-MS) (McKenzie et al., 2018). Despite the accuracy and sensitivity of these techniques, there are some drawbacks, including: not being sufficiently portable, destructive, time-consuming, requiring skilled personnel, and being expensive to run and maintain. Therefore, the use of vibrational techniques such as infrared (Schulz et al., 2004; Risoluti et al., 2016) and Raman spectroscopy (Penido et al., 2016), and surface-enhanced Raman scattering (SERS) (Yu et al., 2018, 2019), has attracted a lot of interest as a reliable alternative (D'Elia et al., 2015) as these physicochemical methods provide a portable (hand-held), rapid and cost-effective solution. In addition to these advantages, these techniques enable on-site quantitative singular or multiplexed detection and identification of xenobiotic compounds such as NPS and their metabolites in human biofluids (Mabbott et al., 2015; Yu et al., 2019). Whilst infrared and Raman spectroscopy have been shown to be valuable tools in forensics, including for the analysis of NPS (Jones et al., 2016), providing detailed chemical information, due to their inherent weak signal, these techniques are perceived to lack the required sensitivity for the detection of compounds at low concentrations (Stiles et al., 2008). Therefore, the specificity and selective sensitivity of SERS promotes this approach as a viable alternative method for such applications, allowing the detection, and absolute quantification of trace quantities of target analytes (Goodacre et al., 2018). Several studies in the literature have reported the application of SERS for quantitative detection of various drugs such as: caffeine (Alharbi et al., 2015b), propranolol (Subaihi et al., 2016), 5,6-methylenedioxy-2-aminoindane (MDAI) (Mabbott et al., 2013), codeine (Subaihi et al., 2017), tramadol (Alharbi et al., 2015a), phenothiazine (Ackermann et al., 2007), and amphetamines (Faulds et al., 2002; Dong et al., 2015; Han et al., 2015).

The primary aim of this study was to demonstrate the application of Raman spectroscopy and SERS combined with chemometrics approaches for the quantitative detection and discrimination of a wide range of NPS (methcathinone and aminoindane derivatives), both as powders and in solution. Furthermore, to examine fully the suitability of this approach as a valid alternative to current methods, a secondary aim was to illustrate the detection and quantification of mephedrone (4-methylmethcathinone) and its metabolites (nor-mephedrone and 4-methylephedrine) in a multiplexed system as tertiary mixtures in water and human urine, in order to translate this approach to real-world applications.

## Materials and Methods

Silver nitrate (99.9% purity), trisodium citrate, and sodium chloride were purchased from Sigma Aldrich (Dorset, United Kingdom). The target compounds (Table S1) were synthesized at Manchester Metropolitan University, under UK Home Office license (No. 337201), and obtained as stable, white to off-white powders. To ensure the authenticity of the materials utilized in this study the synthesized samples were structurally characterized and the purity of all samples was confirmed by NMR and GC-MS (>99.5% in all cases). Stock solutions (0.1 M) of the samples (S1–S16) were prepared in de-ionized (DI) water. Samples S17–S23 did not dissolve in water, thus only Raman spectra of the solid (powder) were collected. Sodium chloride stock solution (0.5 M) was prepared by dissolving 0.29 g NaCl in 10 mL of DI water.

Mid-stream first morning urine samples were collected in 50 mL Falcon tubes. Samples were briefly kept at ~4°C immediately following collection, then transported to the laboratory and stored at −80°C within 2 h of collection. Prior to analysis, samples were allowed to thaw, centrifuged at 5,000 *g* for 10 min at 4°C and any pellet removed before being used.

Ethical Approval for collection of urine from a single healthy volunteer was not required and this is in line with local legislation and is also in line with the 1964 Declaration of Helsinki.

### Preparation and Characterization of Nanoparticles

All glassware were carefully washed in aqua regia (HNO_3_:HCl 1:3) and deionised water and air-dried at room temperature before nanoparticle preparation.

Citrate-reduced Ag colloid was prepared by following the Lee and Meisel (Lee and Meisel, 1982), method. Briefly 90 mg of silver nitrate was dissolved in 500 mL deionised water and heated to boiling. 10 mL of 1% w/v trisodium citrate aqueous solution was then added to the boiling silver nitrate solution, and left stirring for 20 min to stabilize nanoparticle sizes. A color change from colorless to milky green indicated successful synthesis of nanoparticles. The colloids were cooled to room temperature and stored in bottles covered in aluminum foil to prevent light degradation.

Borohydride-reduced Ag colloid (Lee and Meisel, 1982; Zeiri et al., 2002). Silver nitrate (0.104 g) was dissolved in DI water (100 mL). A solution of sodium borohydride (0.057 g) was prepared. Both solutions were cooled separately in ice for 30 min. The ice-cold silver nitrate solution was added dropwise to the sodium borohydride solution with stirring for 20 min. A color change from colorless to a dark milky yellow was observed. Once cool, the flask was wrapped in foil to prevent light degradation.

Silver colloid by hydroxylamine reduction (Leopold and Lendl, 2003). Stock solutions of silver nitrate (0.176 g in 10 mL DI water), sodium hydroxide (0.125 g in 100 mL DI water) and hydroxylamine hydrochloride (1.059 g in 10 mL DI water) were prepared. Sodium hydroxide solution (20 mL) was added to DI water (158 mL) and the resulting solution heated with stirring. Hydroxylamine hydrochloride solution (2 mL) was then added. In a separate flask, silver nitrate solution (2 mL) was diluted with DI water (20 mL) and added dropwise to the sodium hydroxide solution with stirring. Once addition was complete, the solution was left stirring for 15 min. A color change from colorless to gray/purple was observed. Following this, the suspension was left to cool and wrapped in foil to prevent light degradation.

UV-Vis characterization was carried out by collecting absorbance spectra over a range of 300–800 nm using a Thermo Biomate 5 spectrophotometer (Thermo Fischer Scientific Inc., Massachusetts, USA).

### Optimisation of SERS Parameters

For optimization purposes, a stock solution of methcathinone hydrochloride (sample 1) was prepared to test the synthesized nanoparticles and to establish the optimal nanoparticles and conditions (various pH and addition of aggregating agent) for acquiring the most reproducible SERS signal.

### Instrumentation

Raman analysis was undertaken using a Renishaw inVia confocal Raman microscope (Renishaw Plc., Gloucestershire, U.K.) equipped with a 785 nm laser. The instrument was calibrated using a silicon plate focused under a ×50 objective, where a static spectrum centered at 520 cm^−1^ for 1 s at 10% power was collected. Spectral data were collected using the WiRE 3.4 software (Galactic Industries Corp. Salem, NH). All spectra were acquired using the laser power adjusted on the sample to ~28 mW.

SERS spectral data of analyte solutions were collected by mixing 100 μL of sample and 200 μL of the nanoparticle solution in a small glass vial, using Delta Nu Advantage portable Raman spectrometer (Laramie WY, USA) equipped with a 785 nm laser. Power density on the sample was adjusted to ~60 mW for 20 s, with three SERS spectral acquisitions per sample. All SERS spectra were collected from a minimum of three replicate samples within 100–1,800 cm^−1^ range, immediately after mixing the solutions in the glass vials.

### Data Analysis

All Raman and SERS spectra were analyzed using MATLAB software (R2015a) (The Math Works Inc., Natick, USA). Data were baseline corrected using asymmetric least-squares (AsLS) algorithms (Eilers, 2003), using an asymmetry parameter of 0.001 and smoothing parameter of 10,000, and auto-scaled. Principal components analysis (PCA) was carried out to reduce the dimensionality of the data, and scores plots generated to investigate the similarities and differences of spectral features (via loadings plots) of the samples. Partial least squares regression (PLSR) (Geladi and Kowalski, 1986; Gromski et al., 2015) was used to generate a series of multivariate regression models using the spectral data (serial dilutions) of the individual samples. The number of latent variables (PLS factors) were selected using cross validation, with subsequent testing on independent hold out data (test sets).

Multiplexed detection of the target analyte (mephedrone hydrochloride) and its phase I metabolites (nor-mephedrone hydrochloride and 4-methylephedrine hydrochloride) in water and human urine was carried out by generating a set of univariate models via regression of the integrated areas of the discriminatory peaks of interest, against the known concentrations of the analytes.

## Results and Discussion

**Figure 1:**
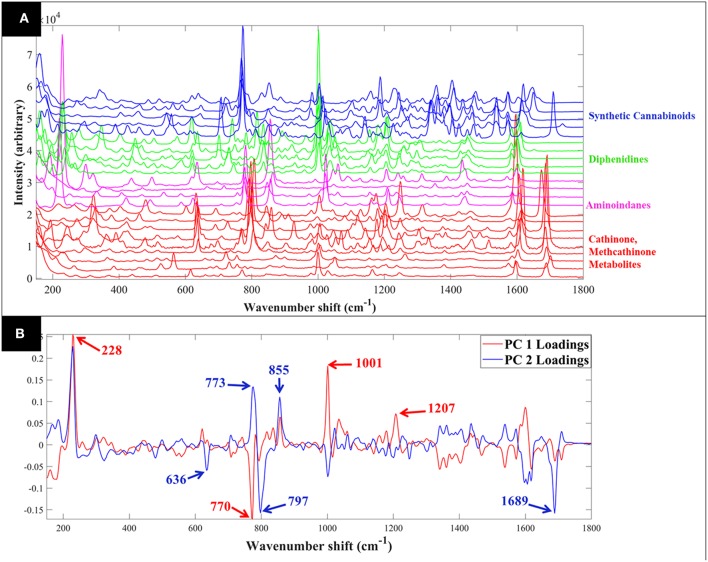
Comparative Raman spectra of all samples collected in solid form **(A)**. PCA loadings plot of the Raman spectral data collected from all samples in solid form **(B)**; this corresponds to the PCA scores plot shown in Figure 3A. Different colors represent the compounds with same core structures (methcathinones in red, aminoindanes in purple, diphenidines in green, and synthetic cannabinoids in blue).

**Figure 2:**
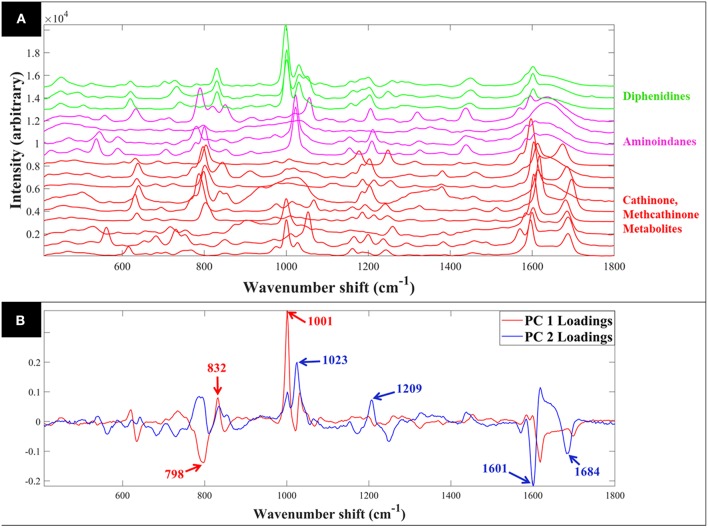
Comparative SERS spectra of all samples collected in aqueous solution **(A)**. PCA loadings plot of the SERS spectral data collected from all samples **(B)**; this corresponds to the PCA scores plot shown in Figure 4. Different colors represent the compounds with same core structures (methcathinones in red, aminoindanes in purple, and diphenidines in green).

**Figure 3:**
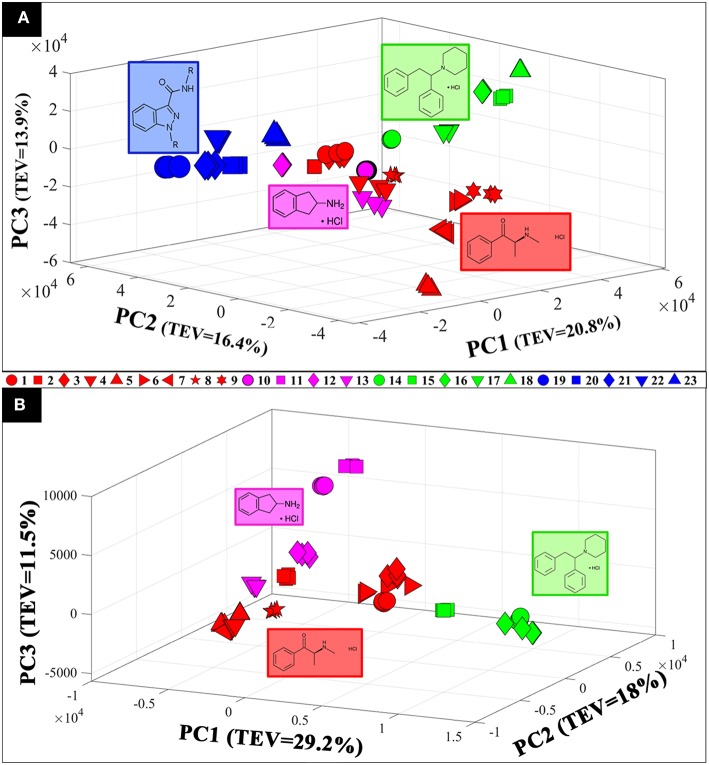
3D PCA scores plots of the Raman spectral data collected from the samples in solid (powder) **(A)** and aqueous form (0.1 M) **(B)**. Different colors represent the compounds with same core structures (methcathinones in red, aminoindanes in purple, diphenidines in green, and synthetic cannabinoids in blue), while the symbols represent the individual samples analyzed in this study. TEV = total explained variance.

Raman spectra of all samples collected in solid (S1–S23: Figure 1A) and liquid (S1–S16: Figure 2A) displayed clear compound-specific vibrational patterns. Multivariate statistical approaches were applied to characterize and differentiate the samples and to see if these were based on core chemical structural differences. PCA was therefore employed to explore these data further (Figure 3). As PCA is an unsupervised learning method any clusters observed would be a natural reflection of any spectral similarities and/or differences. The PCA scores plot of the pre-processed (AsLS and auto-scaled) Raman spectral data collected from the solid (powder) samples (Figure 3A) did indeed display a clear separation of the samples according to their core structures. Whilst the diphenidines (samples 14–18) were separated from all other samples according to PC1 axis (Figure 3A) with a total explained variance (TEV) of 20.8%, the synthetic cannabinoids were clustered on the positive side of PC2, with a TEV of 16.4%, and away from all other samples. According to the PC1 loadings plot of the solid samples (Figure 1B), the most significant vibrational band contributing to the separation of the diphenidines from all other NPS samples was the peak at 1,001 cm^−1^ which can be assigned to aromatic ring breathing/in-plane C-H bending. This is also apparent in the Raman spectra (Figure 1A) exhibiting a sharp peak in this region for all the diphenidines, which is perhaps not surprising considering the structure of this group of compounds containing multiple aromatic rings. In addition, the PC2 loadings plot (Figure 1B) identified the 773 cm^−1^ peak, corresponding to N-N asymmetric stretching of indazole (Islam et al., 2018), to be the most significant feature discriminating between the synthetic cannabinoids and all other samples.

PCA scores plot of the Raman spectral data collected from the samples in solution (Figure 3B) displayed similar clustering patterns, where the diphenidines were again separated from all other samples according to PC1 axis with a TEV of 29.2%, while PC3 allowed for further discrimination of the aminoindanes from methcathinones. The most significant vibrational bands highlighted by the PCA loadings plot (Figure 2B) of these samples were also in agreement with those from the solid samples (Figure 1B).

In order to establish the required optimal condition for achieving the SERS signal, methcathinone hydrochloride (sample 1) and Rhodamine 6G (0.2 mM) was tested with different nanoparticles (silver borohydride, silver citrate, and silver hydroxylamine reduced) in NaCl as the aggregation agent at a range of pH levels (3, 5, 7, and 9). Comparison of the SERS spectra acquired from the three tested nanoparticles demonstrated that the silver hydroxylamine reduced nanoparticles provided the most reproducible spectra, and that the addition of the aggregation agent did not improve the signal (Figures S1A–C, S2). Hence, hydroxylamine-reduced Ag nanoparticles without the addition of an aggregation agent was taken forward and employed as the optimum SERS substrate. To determine the optimal pH for SERS analysis, SERS spectra of methcathinone hydrochloride (sample 1) was collected at different pH levels and the results suggested pH 7 as the optimum condition (Figure S1D).

**Figure 4:**
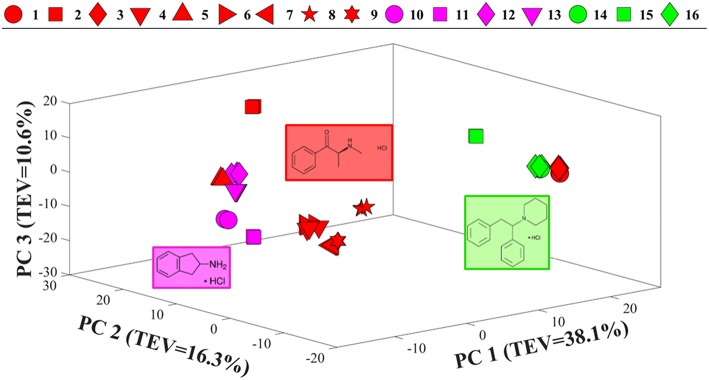
PCA scores plot of all SERS spectral data. Different colors represent the compounds with same core structures (methcathinones in red, aminoindanes in purple, and diphenidines in green), while the symbols represent the individual samples analyzed in this study. TEV = total explained variance.

Comparison of the SERS spectra of all 16 samples in solution (Figure S3) also demonstrated clear vibrational patterns specific to each of the psychoactive compounds. PCA scores plot of these SERS data (Figure 4), were consistent with previous Raman findings (Figure 3), and allowed for the separation of diphenidines from all other samples according to PC1 axis with 38.1% TEV. Surprisingly, two of the methcathinone samples (1 and 3) also clustered closely with the diphenidines. PC1 loadings plot of these data (Figure S4), highlighted the vibrational band at 1,028 cm^−1^ (C-H in plane bending, aromatic stretch) to be the most significant peak distinguishing between the diphenidines and the rest of the samples. This finding also explains the reason for clustering of the two methcathinones (samples 1 and 3) with the diphenidines, as these are the only methcathinone samples which also displayed a strong vibrational band at 1,028 cm^−1^ (Figure S3).

In order to determine the limit of detection (LOD) for each of the compounds via SERS, we followed the procedure as described in Clayton et al. (1987), Ortiz et al. (2003). Dilution series of the individual compounds was prepared in water (Table 1), and the SERS spectra were collected (Figure S5). The PLSR models were built to predict the concentrations of each compound using corresponding SERS spectra. The models were validated using a double cross-validation procedure as described in Westerhuis et al. (2008). The PLSR models displayed acceptable (0.48–0.94) coefficient of determination of validation (*Q*^2^). Based on the predictions of these PLS-R models the estimated LODs varied from the lowest of ~2 mM to the highest of 51 mM for samples 13 and 12, respectively. It is also worth noting that, a comparison of all spectral data collected from the samples in solution displayed clear differences between the Raman and SERS vibrational features and relative intensities, which suggests that the spectra collected from the NPs mixtures are indeed SERS signal.

**Table 1:** Estimated limits of detection (LOD) of all the compounds using SERS.

**Sample No**.	**Conc. range*I*mM**	**LOD/mM**	***Q*2 of double-CV**
1	50.0–3.1	4.25	0.77
2	25.0–1.6	6.05	0.73
3	100.0–3.1	5.62	0.81
4	50.0–1.6	2.44	0.93
5	50.0–3.1	2.99	0.78
6	100.0–6.3	19.13	0.60
7	50.0–1.6	2.45	0.89
8	100.0–1.6	2.21	0.94
9	100.0–3.1	3.37	0.88
10	100.0–1.6	10.94	0.70
11	50.0–3.1	3.21	0.87
12	100.0–3.1	51.01	0.48
13	50.0–1.6	1.99	0.94
14	25.0–1.6	3.60	0.82
15	25.0–1.6	2.46	0.77
16	100.0–1.6	30.86	0.62

*Q2 indicates the prediction-linearity of the PLSR models generated for each of the compounds, from test cross validation samples (i.e., samples not used in the calibration of the PLS models)*.

The above findings clearly demonstrated the potential application of SERS for the detection of various psychoactive drugs using their intrinsic spectral signatures. However, in a real biological system many of these xenobiotic compounds could be metabolized, thus detection of the target compound as well as its metabolites may play a crucial role in monitoring, since these metabolites may still be present after the NPS is no longer detectable. This would also enable a better understanding of the pharmacokinetics and pharmacodynamics of these drugs. To give an example, various studies have reported the potential phase I metabolic pathway of mephedrone (4-MMC) as: (i) N-demethylation of the primary amine to form normephedrone; (ii) reduction of the β-keto moiety to the respective alcohol named dihydromephedrone; and (iii) oxidation of the tolyl moiety to the corresponding alcohol, 4-hydroxytolylmephedrone which undergoes further oxidation (Khreit et al., 2013; Pozo et al., 2015; Elbardisy et al., 2019).

As 4-MMC (sample 4) and two of its main metabolites, nor-mephedrone (Sample 7) and 4-methylephedrine (sample 8), were successfully detected in this study with reasonable LOD values (Table 1), multiplexed detection of these compounds in both water and urine were investigated. In order to mimic the pharmacokinetics of 4-MMC in a real biological system, and based on the reported findings in the literature (Green et al., 2014; Olesti et al., 2017), it was assumed that metabolism to nor-mephedrone was twice that of the metabolism to 4-methylephedrine throughout the experiment. Two sets of samples were prepared in water and human urine, where the concentration of 4-MMC was decreased while the concentration of nor-mephedrone and 4-methylephedrine were gradually increased to mimic a real-life scenario in a biological system (Table S2). Thus, the sample numbers were referred to as time-points to assist such visualization. It is also worth noting that the combined overall concentration of all three compounds in each sample was kept constant.

**Figure 5:**
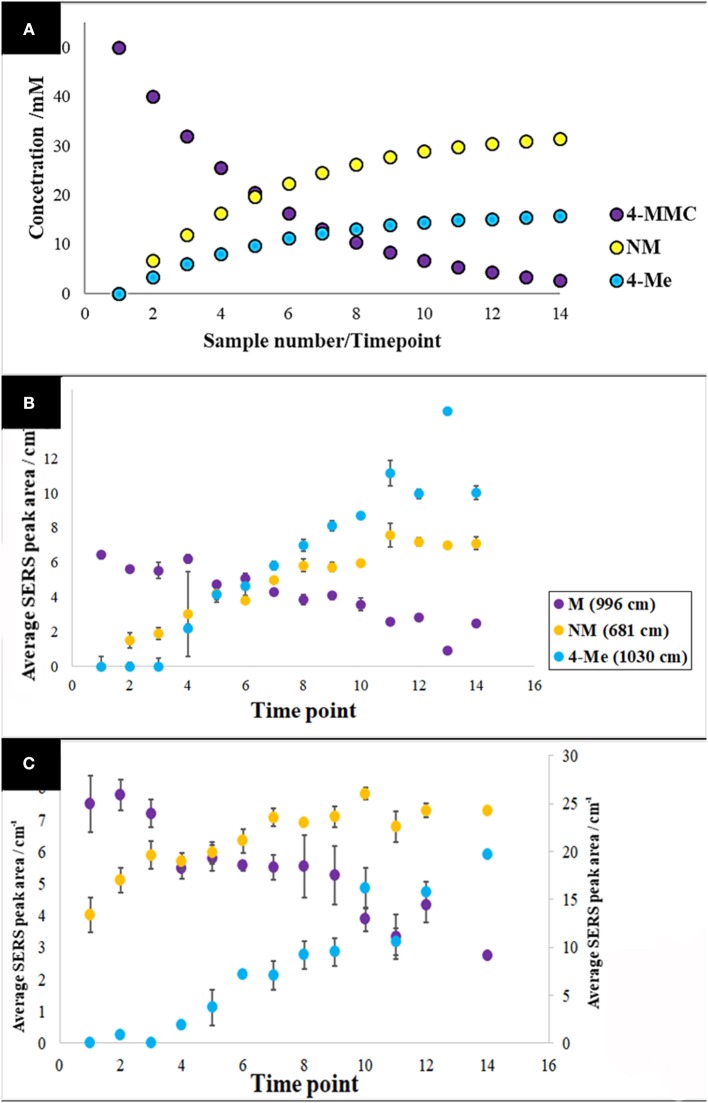
Pharmacokinetic mock-up model for 4-MMC and two of its major metabolites **(A)**. Average metabolite-specific SERS peak areas *vs*. timepoints for the multiplex detection experiment carried out in water **(B)**, and urine **(C)**. The letters 4-MMC, NM, and 4-Me, represent 4-methylmethcathinone, and its metabolites nor-mephedrone and 4-methylephedrine, respectively. The secondary y-axis indicates the detected peak area for NM. Data points presented are average values of three replicates, with error bars indicating the standard deviation.

**Figure 6:**
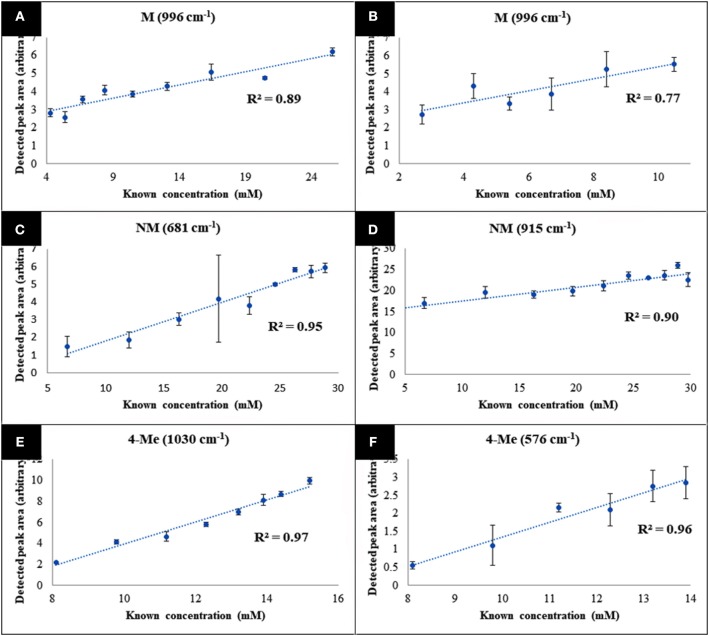
Average metabolite-specific peak areas *vs*. known concentration for the multiplex detection experiment carried out in water **(A–C)**, and urine **(D–F)**. The letters 4-MMC, NM, and 4-Me, represent 4-methylmethcathinone, and its metabolites nor-mephedrone and 4-methylephedrine, respectively. Data points presented are average values of three replicates, with error bars indicating the standard deviation.

The specific vibrational bands for each of the metabolites were identified (Figure S6), and their corresponding peak areas in both water and urine samples were plotted against time-points (sample numbers) (Figure 5). These results clearly displayed the decrease of 4-MMC levels, while its two metabolites exhibited an increasing trend with time. In addition, the known concentration of 4-MMC and its metabolites in the solutions were also plotted against the detected peak areas to examine the linearity of the SERS signal (Figure 6). Although the SERS signal of 4-MMC did not display a linear trend at higher concentrations in the urine samples (>13.1 mM) (Figure 6D), probably due to the complexity of the background matrix (urine) causing saturation of the nanoparticles, all other samples exhibited a linear correlation with a correlation coefficient (*R*^2^) in the range of 0.77–0.97.

## Conclusion

The emergence of NPS, such as cathinones, tryptamines, synthetic cannabinoids, and piperazines, and the vast range of potentially serious side-effects associated with these substances (Hillebrand et al., 2010), illuminates the importance of having rapid, quantitative testing, and detection analytical methods. Thus, the aim of this study was to demonstrate the application of Raman spectroscopy and SERS for quantitative detection and discrimination of a range of NPS as powders and in solution. The detailed and specific vibrational signatures acquired from the Raman and SERS spectral data allowed for clear PCA clustering and separation of the investigated compounds according to their core chemical structures and suggesting that classification would be feasible. To our knowledge this is the first time that multivariate statistics has been applied to Raman or SERS data from NPS that reveal core chemical structures and this may be useful when new NPS are synthesized that have not been detected before as they should cluster with their core structural motif.

The SERS analysis provided LODs mostly ranging between 2.0 and 5.6 mM. Although values in this range are low enough to compare favorably with some techniques currently being used in drug detection, such as microcrystalline testing, they are perhaps not low enough to compare with the current gold standard laboratory techniques such as GC-MS. However, SERS does have the advantage of providing accurate and reliable data rapidly (1 min sample analysis time), with the considerable bonus of the potential of using portable and handheld technologies that could be deployed for on-site testing of samples. Be that point-of-care within clinical settings or in-field testing/identification wherever these psychoactive substances are found. Finally, the successful multiplexed quantitative detection of mephedrone (4-MMC) and two of its main phase I metabolites, nor-mephedrone and 4-methylephedrine, by SERS in both water and human urine, demonstrates further the potential application of this approach for direct detection and quantification of multiple target analytes in human biofluids.

## Data Availability

The raw data supporting the conclusions of this manuscript will be made available by the authors, without undue reservation, to any qualified researcher.

## Author Contributions

HM, OS, and RG designed the experiment. OS generated samples. HM and CJ undertook Raman. HM, AW, MC, AS, and CJ undertook SERS. HM and YX undertook data analysis. All interpreted the data. HM, YX, MC, AS, DE, OS, and RG wrote the manuscript.

### Conflict of Interest Statement

The authors declare that the research was conducted in the absence of any commercial or financial relationships that could be construed as a potential conflict of interest.
