# Supplementary material for: Rapid Detection and Quantification of Novel Psychoactive Substances (NPS) Using Raman Spectroscopy and Surface-Enhanced Raman Scattering
Source: Front Chem. 2019 Jun 19;7:412. doi: 10.3389/fchem.2019.00412 (PMC6593286; doi:10.3389/fchem.2019.00412)
Supplement: Supplementary file 1 [file Data_Sheet_1.docx]

**Rapid detection and quantification of Novel Psychoactive Substances (NPS) using Raman spectroscopy and surface-enhanced Raman scattering**

Howbeer Muhamadali ^1,2^, Alexandra Watt ^2^, Yun Xu ^1,2^, Malama Chisanga ^2^, Abdu Subaihi^2,3^, Carys Jones ^2^, David I. Ellis ^2^, Oliver B. Sutcliffe ^4^, and Royston Goodacre ^1,2^

^1^ Department of Biochemistry, Institute of Integrative Biology, University of Liverpool, Biosciences Building, Crown Street, Liverpool L69 7ZB, UK

^2^ School of Chemistry, Manchester Institute of Biotechnology, University of Manchester, Manchester, M1 7DN, UK

^3^ Department of Chemistry, University College in Al-Qunfudah, Umm Al-Qura niversity,1109 Makkah Al-Mukarramah, Saudi Arabia

^4^ MANchester DRug Analysis and Knowledge Exchange (MANDRAKE), School of Science and the Environment, Faculty of Science and Engineering, Manchester Metropolitan University, John Dalton Building, Chester Street, Manchester, M1 5GD, UK

**Keywords:** Spectroscopy, Raman, SERS, Drug detection, psychoactive compounds

**Table S1.** All samples and their corresponding sample numbers, showing alternative names and structures for each of the psychoactive compounds analysed in this study.

| Samples | Compound name | Synonyms | Structure |
| --- | --- | --- | --- |
| 1 | Methcathinone hydrochloride | MC.HCl | 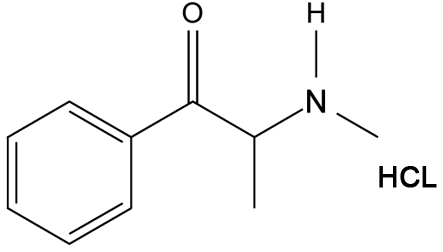 |
| 2 | 2-Methylmethcathinone hydrochloride | 2-MMC.HCl | 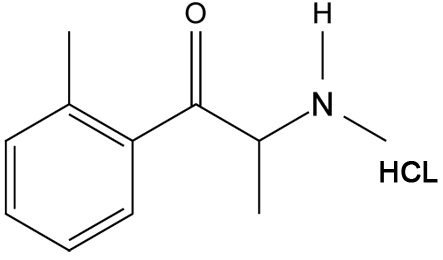 |
| 3 | 3-Methylmethcathinone hydrochloride | 3-MMC.HCl | 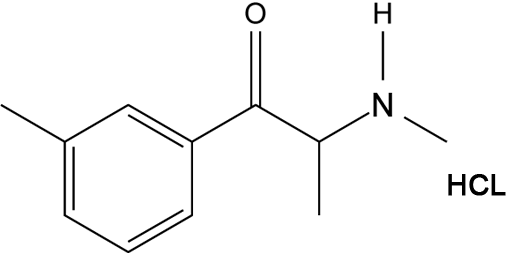 |
| 4 | 4-Methyl-  methcathinone hydrochloride | 4-MMC.HCl | 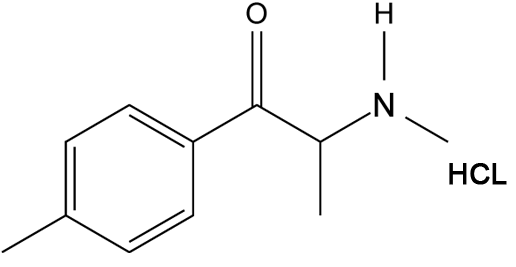 |
| 5 | 4-(Trifluoromethyl)methcathinone hydrochloride | 4-TFMMC.HCl | 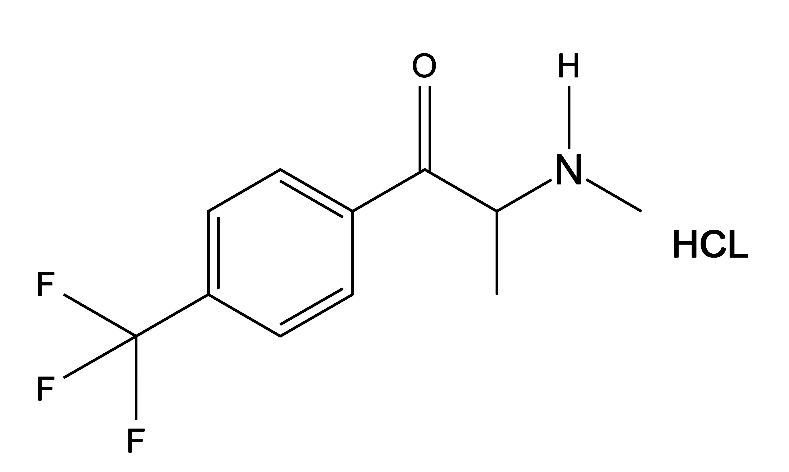 |
| 6 | 4-Methoxymethcathinone hydrobromide | Methedrone.HBr | 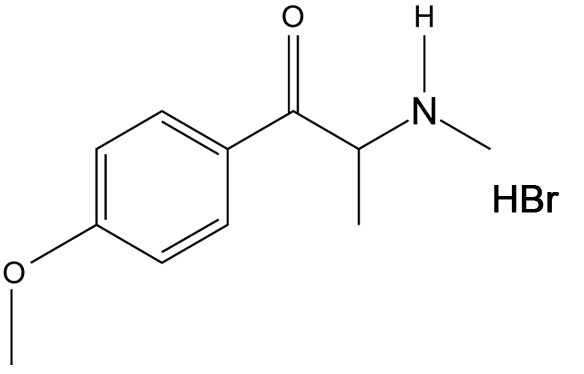 |
| 7 | 4-Methylcathinone hydrochloride | nor-mephedrone.HCl | 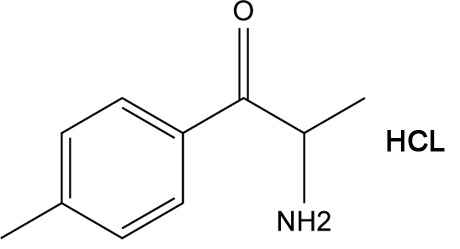 |
| 8 | 4-Methylephedrine hydrochloride | - | 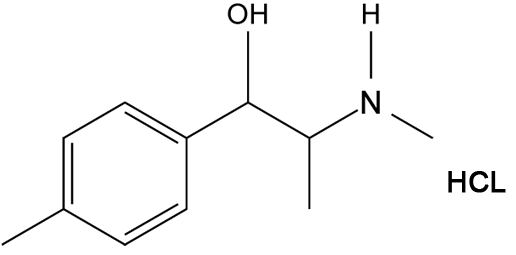 |
| 9 | 4-Methylcathine hydrochloride | - | 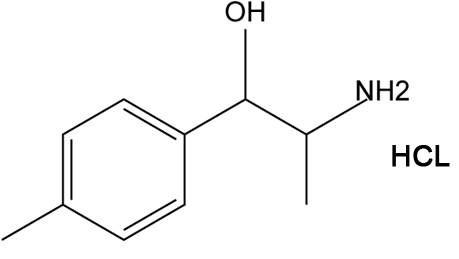 |
| 10 | 2-Aminoindane hydrochloride | 2AI | 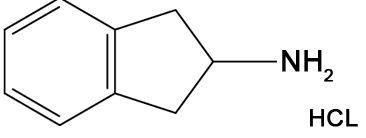 |
| 11 | N-Methyl-2-aminoindane hydrochloride | NM-2AI | 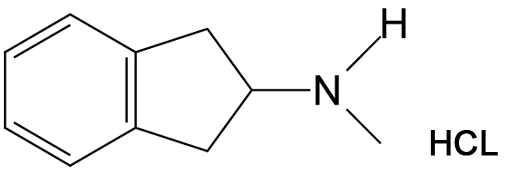 |
| 12 | 5,6-Methylenedioxy- 2-aminoindane hydrochloride | MDAI.HCl | 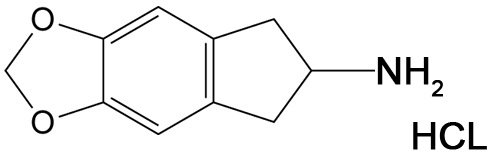 |
| 13 | 5-Iodo-2-aminoindane hydrochloride | 5-IAI.HCl | 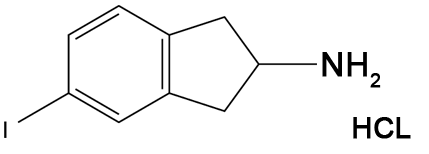 |
| 14 | Diphenidine hydrochloride | DPH.HCl | 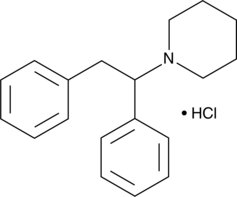 |
| 15 | 2-Methoxphenidine hydrochloride | 2-MXP.HCl | 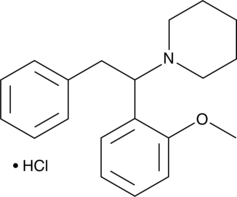 |
| 16 | 3-Methoxphenidine hydrochloride | 3-MXP.HCl | 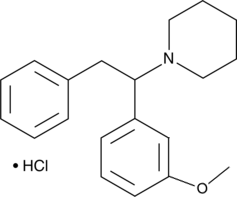 |
| 17 | 4-Methoxphenidine hydrochloride | 4-MXP.HCl | 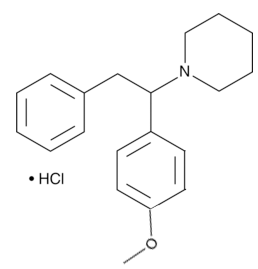 |
| 18 | Ephenidine hydrochloride | EPE | 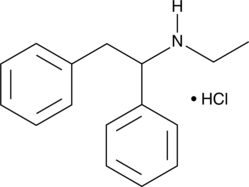 |
| 19 | Isoquinolin-8-yl 1-(cyclohexylmethyl)-1H-indole-3-carboxylate | BB-22 | 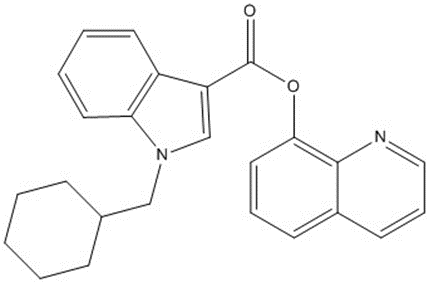 |
| 20 | methyl (S)-2-(1-(cyclohexylmethyl)-1H-indole-3-carboxamido)-3,3-dimethylbutanoate | MMB-CHMINACA | 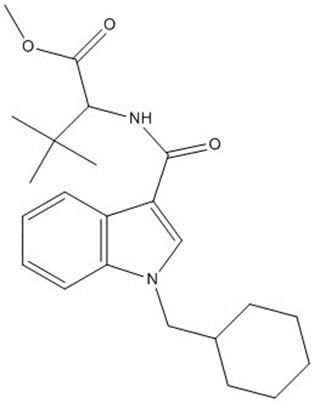 |
| 21 | N-adamantyl-1-fluoropentylindole-3-Carboxamide | STS-135 | 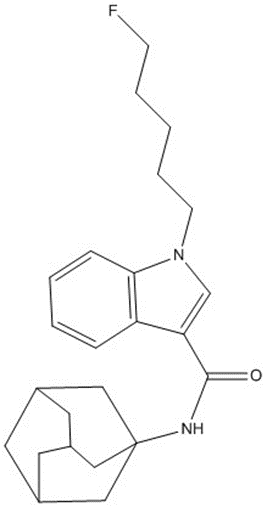 |
| 22 | N-(Adamantan-1-yl)-1-(5-fluoropentyl)-1H-indazole-3-carboxamide | 5F-AKB48 | 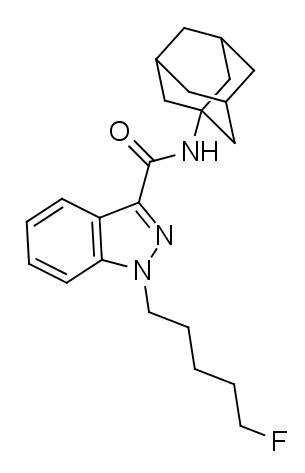 |
| 23 | N-[(1S)-1-(aminocarbonyl)-2-methylpropyl]-1-[(4-fluorophenyl)methyl]-1H-indazole-3-carboxamide | AB-FUBINACA | 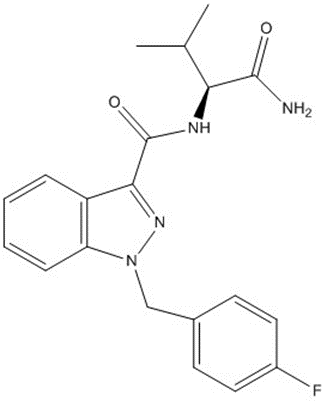 |


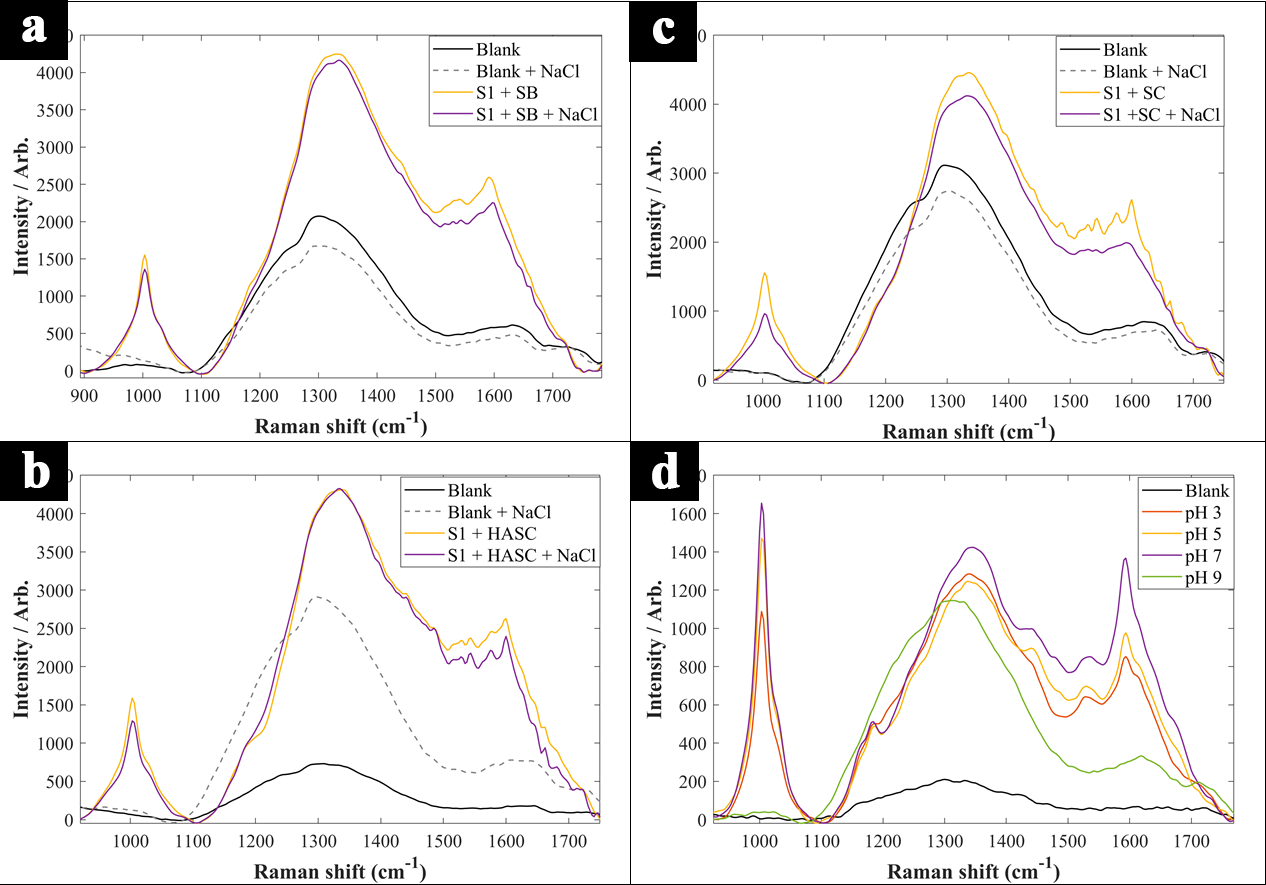


**Figure S1**. Comparison of SERS spectra of sample 1 (S1: methcathinone) generated using different nanoparticles and pH levels. methcathinone with silver borohydride (SB) (+NaCl) **(a)**, methcathinone with silver citrate (SC) (+NaCl) **(b)**, methcathinone with silver hydroxylamine (HASC) (+NaCl) **(c)**, methcathinone with silver hydroxylamine **(d)**. Blank samples are the nanoparticles only.


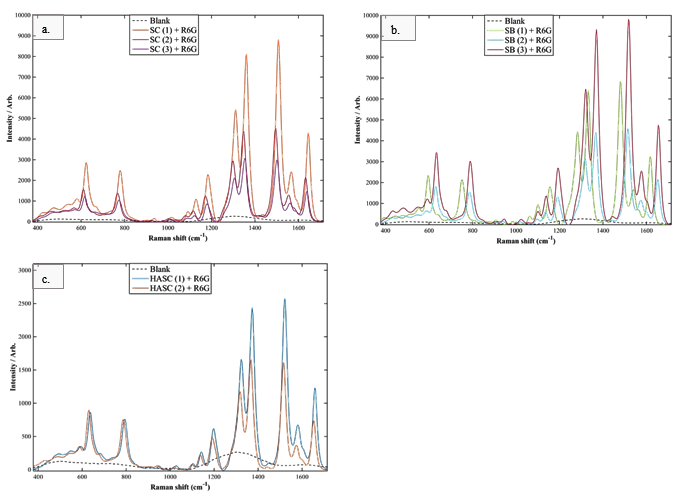


**Figure S2.** Comparison of SERS spectra of R6G acquired using the synthesized colloids; (a) silver citrate reduced colloid, (b) silver borohydride reduced colloid, (c) silver hydroxylamine reduced colloid.


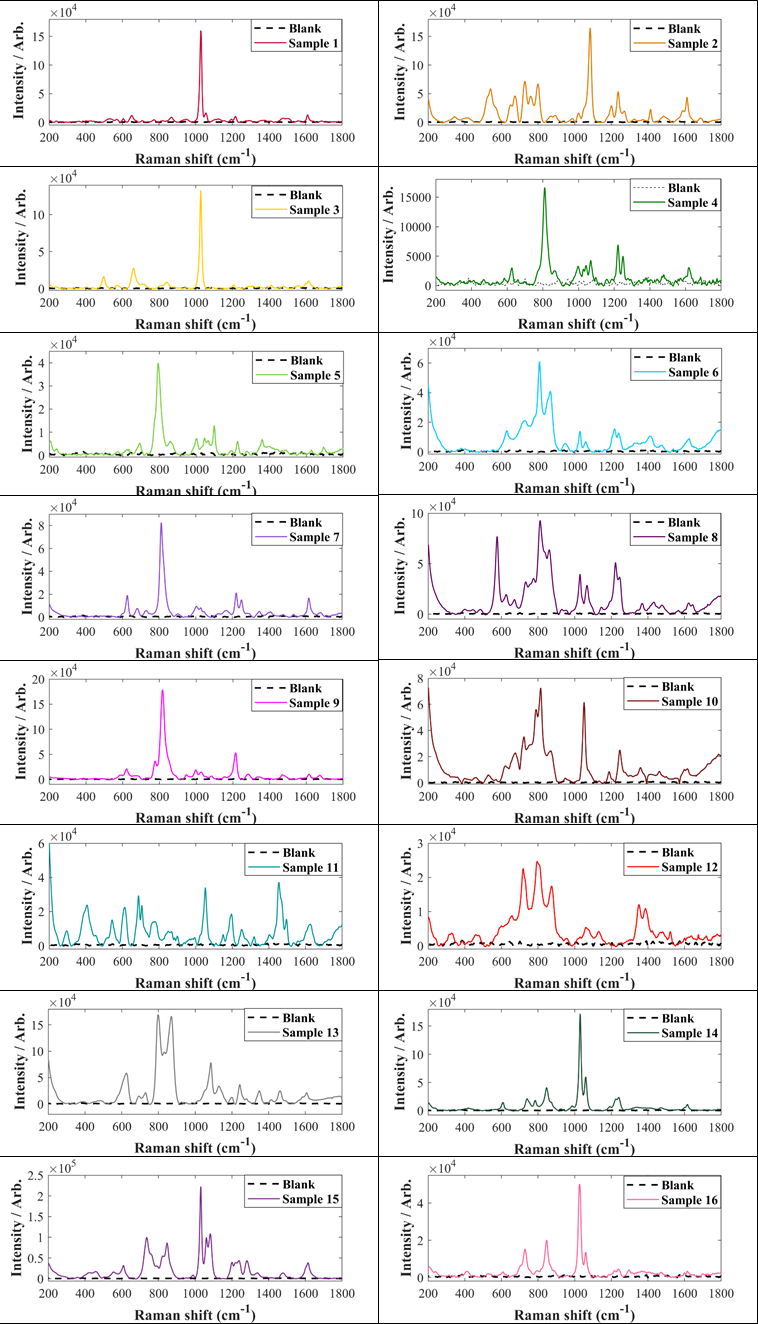


**Figure S3.** Comparison of the SERS spectra of all the samples in liquid form.


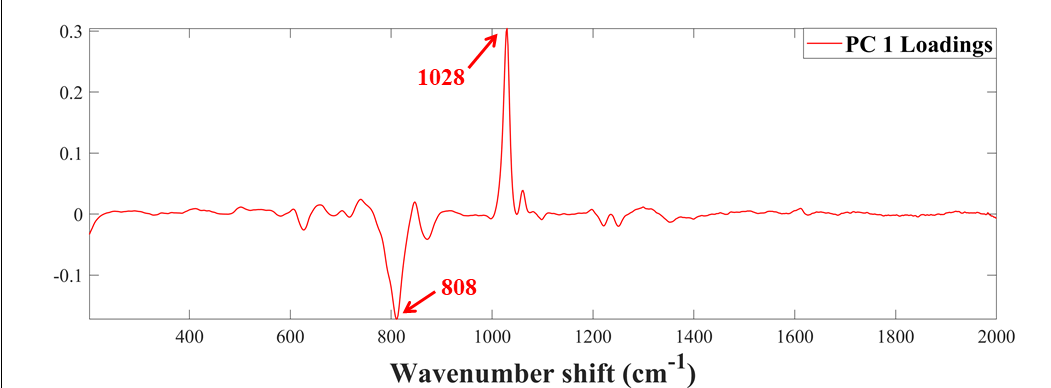


**Figure S4**. PCA loadings plot of all SERS spectral data (this corresponds to the PCA scores plots in the main paper: Fig 4), highlighting the most significant vibrational bands contributing to the clustering patterns in PC1.


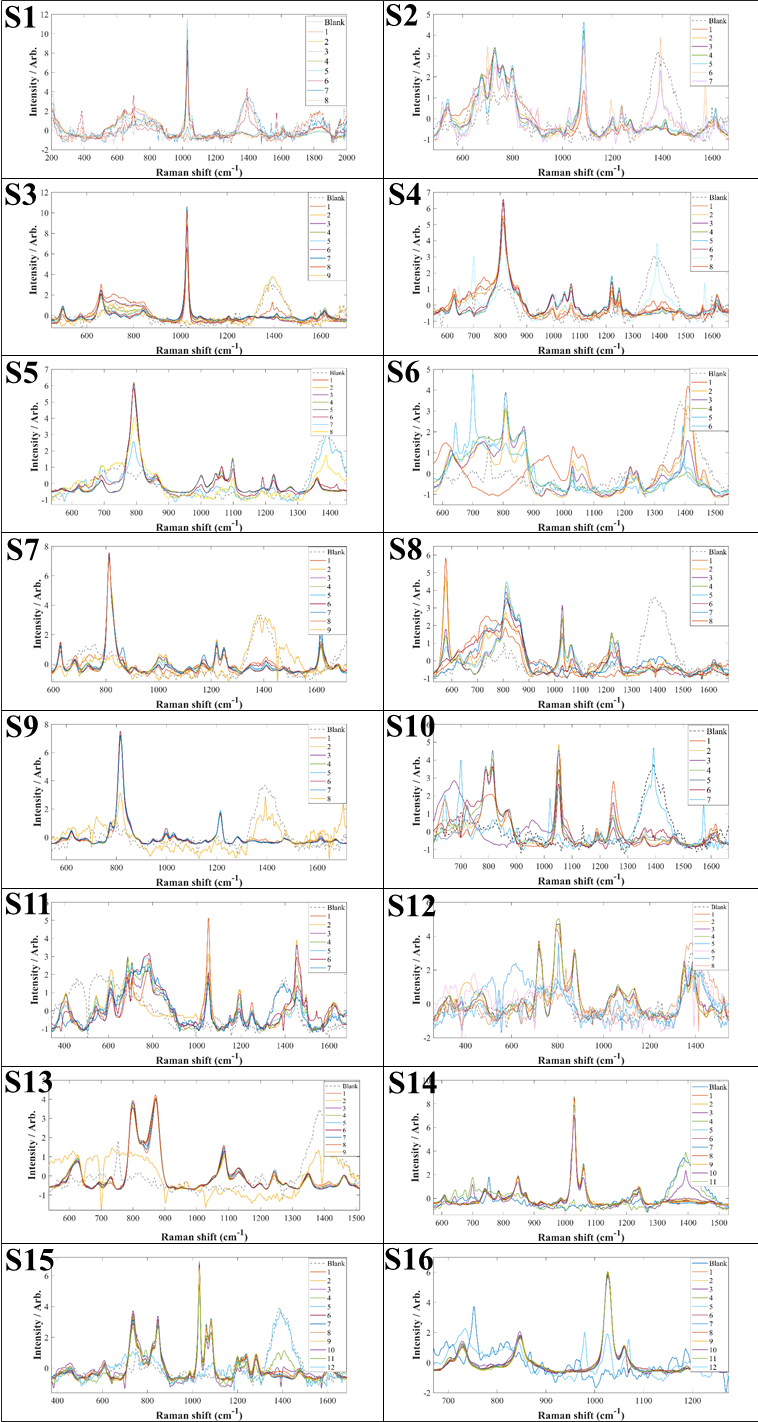


**Figure S5**. Comparison of the SERS spectra generated for all the samples at different concentration ranges, in liquid form. The corresponding concentration ranges for these figures are provided in Table 1.


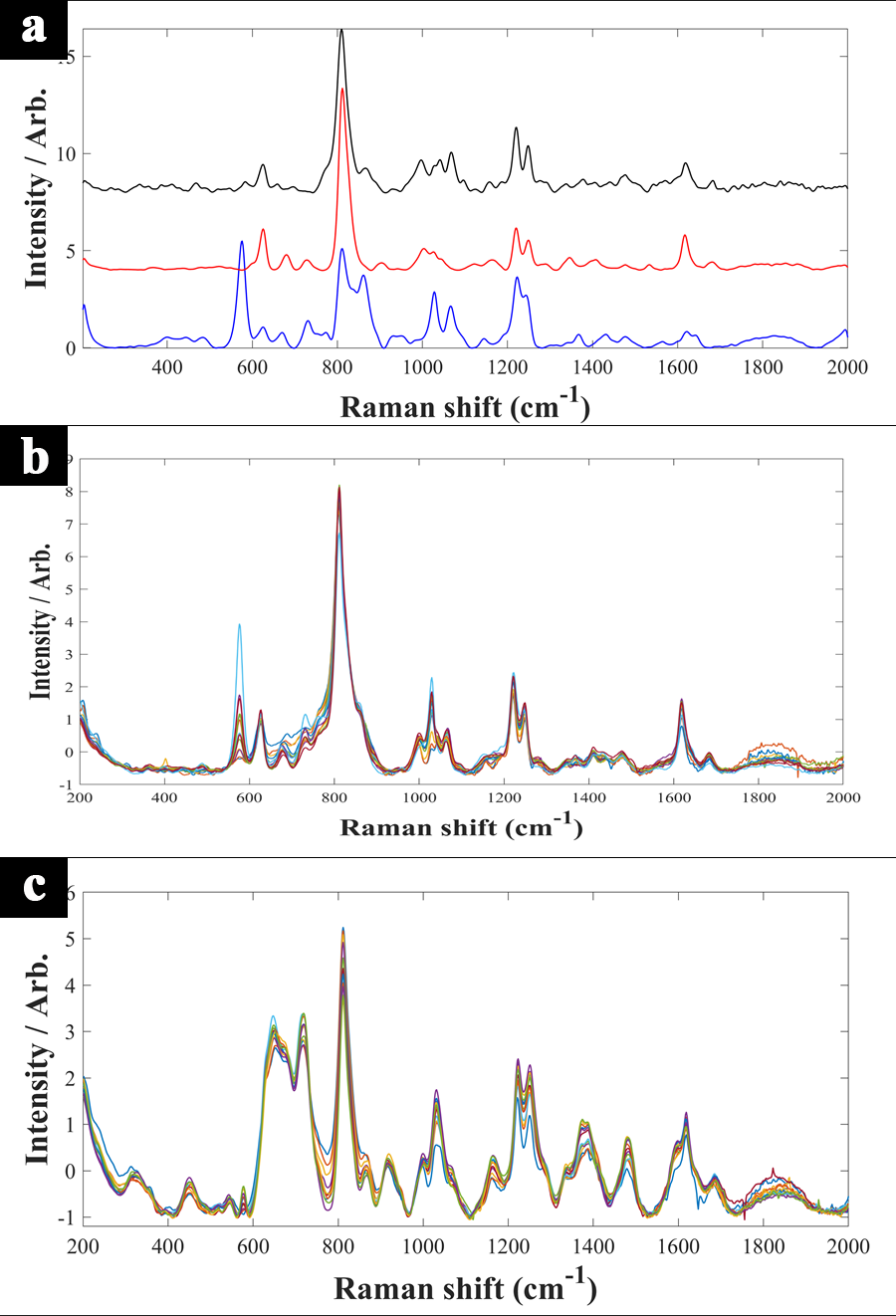


**Figure S6.** Comparison of SERS spectra of 4-MMC (black) and two of its metabolites, nor-mephedrone (red) and 4-methylephedrine (blue) in water **(a)**. Multiplexed SERS spectra of all the samples in water **(b)**, and urine **(c)**.

**Table S2.** Summary of concentration ranges used for the multiplexed detection of 4-MMC and its metabolites in both water and urine.

| Time | 4-MMC | nor-mephedrone | 4-methylephedrine |
| --- | --- | --- | --- |
| 1 | 50 | 0 | 0 |
| 2 | 40 | 6.7 | 3.3 |
| 3 | 32 | 12 | 6 |
| 4 | 25.6 | 16.3 | 8.1 |
| 5 | 20.5 | 19.7 | 9.8 |
| 6 | 16.4 | 22.4 | 11.2 |
| 7 | 13.1 | 24.6 | 12.3 |
| 8 | 10.5 | 26.3 | 13.2 |
| 9 | 8.4 | 27.7 | 13.9 |
| 10 | 6.7 | 28.9 | 14.4 |
| 11 | 5.4 | 29.8 | 14.9 |
| 12 | 4.3 | 30.5 | 15.2 |
| 13 | 3.4 | 31 | 15.5 |
| 14 | 2.7 | 31.5 | 15.8 |
